# The clinical characteristics and underlying causes of liver damage in patients with COVID-19 infection: Retrospective analysis

**DOI:** 10.12669/pjms.37.5.4161

**Published:** 2021

**Authors:** Guojin Zuo, Weirong Liu

**Affiliations:** 1 Guojin Zuo, Department of Ophthalmology, The First Affiliated Hospital of Yangtze University, Jingzhou, Hubei, 434000, China; 2 Weirong Liu Department of Pathology, Yangtze University Health Science Center, Jingzhou, Hubei, 434023, China

**Keywords:** Clinic characteristics, Causes, Liver damage, COVID-19, Retrospective analysis

## Abstract

**Objectives:**

2019-nCoV has become a global threat to human health. The primary objective of this study was to examine the hepatic damage in 2019-nCoV infected patients and the associated underlying causes.

**Methods:**

In this retrospective study, a total of 68 laboratory-confirmed and 20 suspected COVID-19 cases from 23^rd^ January 2020 to 15^th^ February 2020 were included. The study was conducted in The First People’s Hospital of Jingzhou, Hubei. SPSS version 23.0. was used for Statistical analysis using the Student’s t-test or Chi-square test. Data was analyzed for the clinical characteristics and underlying causes of liver damage. The outcomes were followed up until March 29, 2020.

**Results:**

Out of the 68 COVID-19 confirmed cases, 51 had an abnormal liver function, of which 15 had an abnormal liver function at the time of hospital admission. The relationship between the liver function and clinical prognosis of patients showed that the abnormal liver function was positively correlated with the severity of the infection (100% vs.70.2%, p=0.036). The proportion of patients with an elevated level of ALT and a depleted level of Albumin (ALB) were significantly lower in the COVID-19 suspected group than the confirmed group (5% vs. 50.9%, p=0.000; 10% vs. 35.8%, p=0.030, respectively). Besides, the utilization rate of lopinavir/ritonavir, azithromycin, and methylprednisolone in COVID-19 suspected patients were significantly lower than the confirmed patients (25% vs. 62.3%, p=0.004; 35% vs. 62.3%, p=0.037; 25% vs. 64.2%, p=0.003, respectively).

**Conclusions:**

Liver function anomalies are one of the common symptoms associated with the COVID-19 infection, where virus-replication in the liver cells, virus-induced inflammatory response, and administration of clinical medication could be the plausible reason.

## INTRODUCTION

In December 2019, pneumonia cases of unknown origin were reported by medical institutions.^1^ Later, the causative agent of this pneumonia was confirmed to be a new coronavirus–2019 novel coronavirus (2019-nCoV), and the infection was termed as 2019 novel coronavirus disease (COVID-19).^2^ Most of the other coronaviruses strains cause either a respiratory or an enteric change. In addition to these symptoms, 2019-nCoV could lead to the dysfunctionality of multiple organs such as the heart, liver, kidney, immune system, and central nervous system.^3^-^5^ This study retrospectively discussed the liver function impairment and clinical treatment of 68 confirmed and 20 suspected COVID-19 patients to investigate the underlying causes of liver malfunction and improve the efficacy of the treatment regime for COVID-19 infected patients.

## METHODS

The patients included in this study were the one hospitalized at The First People’s Hospital of Jingzhou, Hubei. This was a retrospective study, and all patients were discharged. The 68 COVID-19 confirmed cases were categorized into normal liver function group (n=17) and abnormal liver function group (n=51). All the 68 patients either belong to the moderately infected group (n=57), or the severely infected group (n=11). Out of the 68 COVID-19 confirmed patients, 15 patients had an abnormal liver function at the time of admission, and the rest of the 53 patients were categorized into the normal liver function group (n=17) and the abnormal liver function group (n=36). Serum hepatic enzymes data and clinical medication records were collected for each of these groups as well as for COVID-19 suspected patients (n=20).

### Ethical Approval

The study was approved by the Institutional Ethics Committee of the First People’s Hospital of Jingzhou (Date: March 29, 2020), and written informed consent was obtained from all participants

### Statistical Analysis

We summarized the categorical variables as counts and percentages. Continuous variables were expressed as medians. All analyses were performed with SPSS software, version 23.0. Statistical analysis included the Student’s t-test or Chi-square test. p<0.05 was considered as statistically significant.

## RESULTS

Out of the 68 confirmed patients, 36 were male, and 32 were female, 36 were ≤50 years old, and 32 were ≤50 years old. All the patients were categorized into normal (n=17) and the abnormal (n=51) liver function groups. The proportion of female patients in the normal liver function group was significantly higher than the abnormal liver function group (88.2% vs. 33.3%, p=0.000). However, no significant differences were observed in age, comorbidities, and fever between the two groups (p>0.05). Routine blood analysis indicated that the proportion of patients with decreased lymphocyte count and lymphocytes percentage in the abnormal liver function group were higher than the normal liver function group; however, the difference was insignificant (p>0.05) (Table-I).

**Table-I:** Clinical characteristics of COVID-19 patients: the normal and abnormal liver function groups.

Clinical characteristics	All (n=68)	Normal liver function (n=17)	Abnormal liver function (n=51)	p
***Sex***				
Male	36 (52.9%)	2 (11.8%)	34 (66.7%)	0.000
Female	32 (47.1%)	15 (88.2%)	17 (33.3%)	
***Age***				
≤50	36 (52.9%)	12 (70.6%)	24 (47.1%)	0.092
>50	32 (47.1%)	5 (29.4%)	27 (52.9%)	0.092
***Comorbidities***				
Hypertension	9 (13.2%)	ND	9 (17.6%)	0.063
Diabetes	2 (2.9%)	ND	2 (3.9%)	0.407
***Clinical manifestations***				
Temperature				
Fever	51 (75%)	12 (70.6%)	39 (76.5%)	0.628
Blood routine				
WBC ↓	12 (17.6%)	4 (23.5%)	8 (15.7%)	0.463
Lymphocyte count↓	37 (54.4%)	8 (47.1%)	29 (50.9%)	0.482
Lymphocyte percentage ↓	28 (41.2%)	5 (29.4%)	23 (45.1%)	0.255

**Fig.1:**
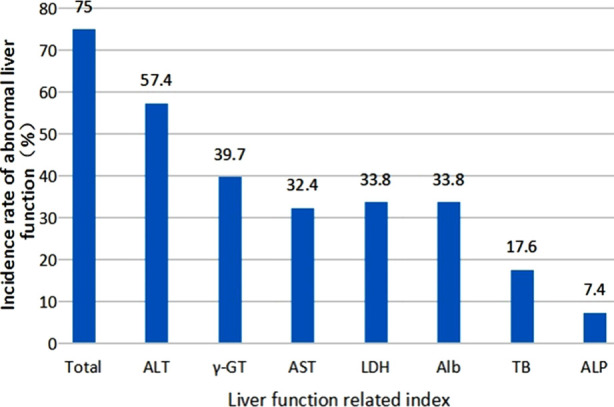
The percentage of abnormal liver function indices.

Out of the 68 COVID-19 confirmed patients, 51 (75%) patients had an abnormal liver function: 15 (29%) at the time of hospital admission and 36 (71%) after the hospital admission. Incidence rates of elevated ALT, Gamma-glutamyl transferase(γ-GT), AST, lactate dehydrogenase (LDH), total bilirubin (TB), and alkaline phosphatase (ALP) were 57.4%, 39.7%, 32.4%, 33.8%, 17.6%, and 7.4%, respectively and the incidence of depleted Alb was 33.8% in the COVID-19 confirmed cases with abnormal liver function (Fig.1).

Excluding those patients with the liver malfunction before hospital admission, the rest 53 confirmed COVID-19 cases were compared with the suspected cases. Abnormal liver function was observed in 50% of the suspected, and 67.9% of the confirmed COVID-19 cases after hospital admission. The proportion of the suspected COVID-19 cases with elevated ALT and reduced Alb levels were significantly lower than the confirmed COVID-19 cases (5% vs. 50.9%, p=0.000; 10% vs. 35.8%, p=0.030, respectively) (Table-II).

**Table-II:** Abnormal liver function in COVID-19 suspected and confirmed cases.

Liver function	COVID-19 suspected cases (n=20)	COVID-19 confirmed cases (n=53)	p
Liver damage	10 (50%)	36 (67.9%)	0.157
Alb↓	2 (10%)	19 (35.8%)	0.030
TB↑	5 (25%)	7 (13.2%)	0.225
ALT↑	1 (5%)	27 (50.9%)	0.000
AST↑	2 (10%)	16 (30.2%)	0.074
γ-GT↑	4 (20%)	19 (35.8%)	0.194
ALP↑	1 (5%)	3 (5.7%)	0.912
LDH↑	4 (20%)	18 (34.0%)	0.246

↓Represents decreased liver enzyme, ↑ represents elevated liver enzyme.

All the confirmed and suspected COVID-19 patients were treated with antivirals and antibiotics. Out of the 68 confirmed COVID-19 patients, 53 patients did not show any sign of liver malfunction prior to the drug administration. However, 36 of the patients developed liver malfunction after hospitai admission. The most common antiviral medications given to these 53 patients were lopinavir/ritonavir and arbidol, and their utilization rates were 62.3% and 77.4%, respectively. It was followed by ribavirin, interferon, chloroquine phosphate/plaquenil, and oseltamivir, their utilization rates were 26.4%, 22.6%, 22.6%, and 18.9%, respectively.

The most common antibiotics administered to these 53 patients were moxifloxacin and azithromycin, and their utilization rates were 83% and 62.3%, respectively, followed by beta-lactam antibiotics (30.2%). There was no significant difference in the antibiotic utilization rate between abnormal and normal liver function groups (P>0.05). The overall utilization rate of methylprednisolone was found to be 64.2%, of which the normal liver function group accounted for 47.1%, and abnormal liver function group accounted for 72.2%. The utilization rate of azithromycin and methylprednisolone in the suspected COVID-19 cases was significantly lower than the confirmed COVID-19 cases (35% vs. 62.3%, p=0.037; 25% vs. 64.2%, p=0.003) (Table-III).

**Table-III:** The medications administered in COVID-19 suspected and confirmed patients with normal or abnormal liver function.

Drugs administered	% of suspected cases (n=20)	% of confirmed COVID-19 cases			P1	P2

		All cases (n=53)	Normal liver function (n=17)	Abnormal liver function (n=36)		
***Antivirals***						
Lopinavir/ritonavir	5 (25%)	33 (62.3%)	10 (58.8%)	23 (63.9%)	0.723	0.004
Arbidol	16 (80%)	41 (77.4%)	11 (64.7%)	30 (83.3%)	0.130	0.808
Ribavirin	6 (30%)	14 (26.4%)	3 (17.6%)	11 (30.6%)	0.320	0.759
Oseltamivir	1 (5%)	10 (18.9%)	4 (23.5%)	6 (16.7%)	0.551	0.140
Interferon	2 (10%)	12 (22.6%)	3 (17.6%)	9 (25%)	0.550	0.221
Chloroquine phosphate/Plaquenil	1 (5%)	12 (22.6%)	2 (20%)	10 (27.8%)	0.194	0.079
***Antibiotics***						
Moxifloxacin	16 (80%)	44 (83.0%)	12 (70.6%)	32 (88.9%)	0.390	0.885
Azithromycin	7 (35%)	33 (62.3%)	12 (70.6%)	21 (58.3%)	0.390	0.037
Beta-lactam	9 (45%)	16 (30.2%)	3 (17.6%)	13 (36.1%)	0.172	0.234
Methyl predisolone	5 (25%)	34 (64.2%)	8 (47.1%)	26 (72.2%)	0.075	0.003

P1 represents the statistical difference between the normal and abnormal liver function groups of 53 confirmed COVID-19 patients, and P2 represents the statistical difference between the COVID-19 suspected and confirmed patients.

### Relationship between the liver function and clinical outcomes

Out of the 68 patients, 51 (75%) patients had an abnormal liver function in which the proportion of patients in abnormal liver function was significantly higher in the group with severe COVID-19 infection than the moderately infected group (100% vs.70.2%; p=0.036). The proportion of patients with elevated ALT, AST, LDH, and decreased Alb levels was significantly higher in the group with severe COVID-19 infection than the moderately infected group (90.9% vs. 50.9%, p=0.014; 90.9% vs. 21.1%, p=0.000; 100% vs. 21.1%, p=0.000; 90.9% vs. 22.8%, p=0.000, respectively) (Table-IV).

**Table-IV:** Liver function indices of patients with moderate or severe COVID-19 infection.

Liver function	All patients (n=68)	Moderately infected (n=57)	Severely infected (n=11)	p
Liver damage	51 (75%)	40 (70.2%)	11 (100%)	0.036
ALT↑	39 (57.4%)	29 (50.9%)	10 (90.9%)	0.014
AST↑	22 (32.4%)	12 (21.1%)	10 (90.9%)	0.000
LDH↑	23 (33.8%)	12 (21.1%)	11 (100%)	0.000
Alb↓	23 (33.8%)	13 (22.8%)	10 (90.9%)	0.000
TB↑	12 (17.6%)	10 (17.5%)	2 (18.2%)	0.959
γ-GT↑	27 (39.7%)	22 (38.6%)	5 (45.5%)	0.670
ALP↑	5 (7.4%)	4 (7.0%)	1 (9.1%)	0.809

↓Represents decreased liver enzyme, ↑ represents elevated liver enzyme.

## DISCUSSION

Severe acute respiratory syndrome coronavirus (SARS-CoV), middle east respiratory syndrome coronavirus (MERS-CoV),^6^ and 2019-nCoV are the three zoonotic and highly pathogenic human coronavirus species that emerged in the 21^st^ century. These three viruses can result in respiratory, intestinal, hepatic, and neuronal disorders. It might also cause acute respiratory distress syndrome (ARDS), multiple organ failure (MOF), and even death in severe cases.^1^,^7^

Liver damage is presented in patients infected with any of these three-human coronaviruses. Recent studies on COVID-19 have shown that the incidence of liver injury affected around 14.8%-53% of cases, indicated by an abnormal ALT/AST levels accompanied by slightly elevated bilirubin levels.^8^ In our study, 75% of the COVID-19 infected patients presented abnormal liver function, which was slightly higher than other reports. It might be due to a small sample size and different liver function indices included in this study.

In the current study, the primary liver injury was indicated by an abnormal ALT level, followed by AST, γ-GT, LDH, and Alb levels, and a small number of patients showed increased TB and ALP levels. These findings were in-line with other reports. Recent studies indicate that the virus, systemic inflammatory response, and drug application might cause abnormal liver function in COVID-19 patients.^9^ In the current study, 15 patients showed abnormal liver function at the time of hospital admission, prior to clinical treatment, which indicates virus-induced liver damage. Similar to the SARS infection, 2019-nCoV binds to the host-cell ACE2 receptor to enter the host cells, which initiates the infection.^10^ As suggested by a preliminary study, the ACE2 receptor expression is enriched in cholangiocytes. These multifunctional cells play a crucial role in liver regeneration and immune response.^11^ In our study, we observed an escalated level of cholangiocyte injury biomarkers, i.e., γ-GT and ALP, in 39.7% and 7.4% of all patients, respectively, which might have been induced by the 2019-nCoV. Nevertheless, viral inclusions could not be demonstrated in the pathological analysis of COVID-19 infected cadaveric liver tissue.^4^ Therefore, the hypothesis that COVID-19 infection might induce liver damage needs to be further validated.

Drug-induced liver injury as one of the underlying causes of liver malfunction in COVID-19 patients warrants further investigation. Similar to the SARS treatment regime, antibiotics, antivirals, and steroids were widely used to treat the COVID-19. These drugs might have led to liver injury in COVID-19 infected patients. As shown by a previous study, moxifloxacin was a drug of choice for COVID-19 treatment, but it was found to be associated with the risk of fulminant hepatitis.^12^ Besides, lopinavir/ritonavir is mainly metabolized by liver CYP3A, leading to elevated serum transaminase levels.^13^ Methylprednisolone is also metabolized mainly by the CYP3A4 enzyme, but with mild hepatotoxicity^14^ and chloroquine showed hypersensitivity and liver damage as well.^15^ In our report, all the 20 suspected patients had a normal liver function before hospital admission and were treated with antivirals and antibiotics after hospital admission, and five of them received steroid therapy. During the treatment, 10 patients developed liver injury of different degrees, which could be drug-related. Excluding those patients with liver malfunction before hospital admission, in the 53 confirmed COVID-19 cases, the proportion of cases with the abnormal liver function after hospital admission was slightly higher than the COVID-19 suspected cases. The treatment status showed that the utilization rate of lopinavir/ritonavir, azithromycin, and steroids in confirmed COVID-19 cases was significantly higher than the suspected cases. Chloroquine and interferon were also administered slightly more than in the COVID-19 suspected cases. These findings indicate that apart from the viruses, the drug could also induce abnormal liver function in COVID-19 confirmed cases than in suspected cases. It suggests that the clinicians should weigh the pros and cons while defining the treatment regime for the COVID-19 patients so as to minimize the damage to the liver.

The virus-induced inflammatory response may be one of the causes of liver damage in COVID-19 patients. Previous reports state that patients with severe pneumonia commonly encounter cytokine storm mediated liver damage. The previous study has found a significant decrease in the T cell counts, specifically CD8 + T cells, coupled with an escalated levels of cytokines such as IL-6, IL-10, IL-2, and IFN-γ levels in the peripheral blood of the patients with severe COVID-19 infection as compared to the moderately infected patients.^16^ The patient’s immune system plays a crucial role in overcoming COVID-19 infection. However, an over activated immune system in an attempt to kill the virus might lead to the production of a myriad of inflammatory cytokines resulting in a severe cytokine storm. This might have a fatal outcome due to organ damage, edema, dysfunctional air exchange, acute respiratory distress syndrome (ARDS), acute cardiac injury, and secondary infection.^17^ In the current study, we observed that abnormal liver function was more common in patients with severe COVID-19 infection. All these cases showed different degrees of abnormal liver function. The proportion of patients with elevated ALT, AST, LDH, and decreased Alb levels were significantly higher in patients with severe COVID-19 infection than patients with moderate COVID-19 infection. These findings are in line with the previous reports.^18^-^20^ However, the role of the inflammatory response in liver damage of COVID-19 infected patients demands further investigation. Mitigation of the cytokine storms through clinical treatment remains crucial in the prognosis of patients with severe COVID-19 infection.

### Strengths and Limitations of this study

This article, for the first time, reported the clinical characteristics and underlying causes of liver damage in the COVID-19 infected and suspected cases from Jingzhou, Hubei. The limitation of the present study is the small cohort that may have induced bias. Due to the absence of supporting laboratory data, the underlying causes of liver injury in COVID-19 infected patients needs further validation.

## CONCLUSIONS

Abnormal liver enzyme activities are commom in patients with COVID-19. Direct virus-induced cytopathic effects, overshooting inflammatory responses and hospital treatment might induce liver damage in COVID-19 patients.

### Authors’ Contributions:

**Guojin Zuo** and **Weirong Liu** designed this study and prepared this manuscript, and are responsible and accountable for the accuracy or integrity of the work.

**Guojin Zuo** and collected and analyzed clinical data.

**Weirong Liu** significantly revised this manuscript.
